# Roles of Reactive Oxygen Species in Anticancer Therapy with* Salvia miltiorrhiza Bunge*

**DOI:** 10.1155/2016/5293284

**Published:** 2016-08-04

**Authors:** Yu-Chiang Hung, Tai-Long Pan, Wen-Long Hu

**Affiliations:** ^1^Department of Chinese Medicine, Kaohsiung Chang Gung Memorial Hospital and Chang Gung University College of Medicine, No. 123, Dapi Road, Niaosong District, Kaohsiung 83342, Taiwan; ^2^School of Chinese Medicine for Post Baccalaureate, I-Shou University, No. 1, Sec. 1, Syuecheng Road, Dashu District, Kaohsiung 84001, Taiwan; ^3^School of Traditional Chinese Medicine, Chang Gung University, No. 259 Wen-Hwa 1st Road, Kweishan, Taoyuan 33302, Taiwan; ^4^Liver Research Center, Chang Gung Memorial Hospital, No. 259 Wen-Hwa 1st Road, Kweishan, Taoyuan 33302, Taiwan; ^5^Research Center for Industry of Human Ecology, Chang Gung University of Science and Technology, Kweishan, Taoyuan 83302, Taiwan; ^6^Department of Medical Research, China Medical University Hospital, China Medical University, No. 91 Hsush-Shih Road, Taichung 40402, Taiwan; ^7^Kaohsiung Medical University College of Medicine, No. 100, Shihcyuan 1st Road, Sanmin District, Kaohsiung 807, Taiwan; ^8^Fooyin University College of Nursing, No. 151, Chinhsueh Road, Ta-Liao District, Kaohsiung 831, Taiwan

## Abstract

Cancer is a leading cause of death worldwide. We aim to provide a systematic review about the roles of reactive oxygen species (ROS) in anticancer therapy with* Salvia miltiorrhiza* Bunge (Danshen). Danshen, including its lipophilic and hydrophilic constituents, is potentially beneficial for treating various cancers. The mechanisms of ROS-related anticancer effects of Danshen vary depending on the specific type of cancer cells involved. Danshen may enhance TNF-*α*-induced apoptosis, upregulate caspase-3, caspase-8, caspase-9, endoplasmic reticulum stress, P21, P53, Bax/Bcl-2, DR5, and AMP-activated protein kinase, or activate the p38/JNK, mitogen-activated protein kinase, and FasL signaling pathways. Conversely, Danshen may downregulate human telomerase reverse transcriptase mRNA, telomerase, survivin, vascular endothelial growth factor/vascular endothelial growth factor receptor 2, CD31, NF-*κ*B, Erk1/2, matrix metalloproteinases, microtubule assembly, and receptor tyrosine kinases including epidermal growth factor receptors, HER2, and P-glycoprotein and inhibit the PI3K/Akt/mTOR or estrogen receptor signaling pathways. Therefore, Danshen may inhibit cancer cells proliferation through antioxidation on tumor initiation and induce apoptosis or autophagy through ROS generation on tumor progression, tumor promotion, and tumor metastasis. Based on the available evidence regarding its anticancer properties, this review provides new insights for further anticancer research or clinical trials with Danshen.

## 1. Introduction

**Figure 1:**
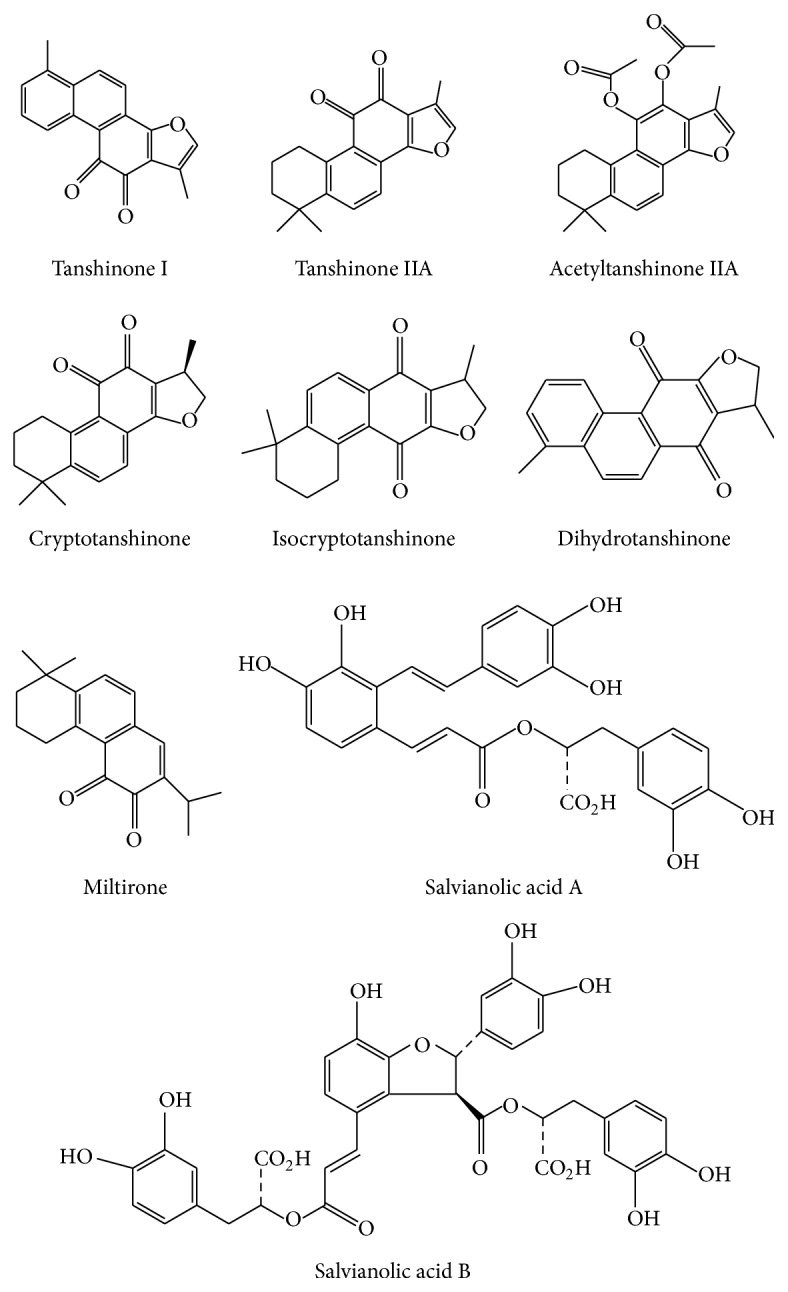
Chemical structures of the different constituents of Danshen. Danshen contains lipophilic compounds including tanshinone I, tanshinone IIA, acetyltanshinone IIA, cryptotanshinone, isocryptotanshinone, dihydrotanshinone, and miltirone. Danshen also contains hydrophilic phenolic acids including salvianolic acids A and B.

Cancer is a leading cause of mortality throughout the world. In addition to conventional therapies such as surgery, chemotherapy, and radiotherapy, traditional Chinese medicine and other complementary or alternative therapies may be necessary for cancer patients [1].* Salvia miltiorrhiza Bunge *(Danshen) has been used widely for the treatment of various diseases [2–7] including cancers [8–12] for thousands of years within the China community. Danshen, a Chinese herbal medicine, contains two major groups of chemicals [12–15]. The first group includes lipophilic compounds such as tanshinone I, tanshinone IIA, acetyltanshinone IIA, cryptotanshinone, isocryptotanshinone, dihydrotanshinone, 15,16-dihydrotanshinone I, and miltirone. The second group includes the hydrophilic phenolic acids such as salvianolic acids A and B (Figure 1). Our research and numerous other publications have demonstrated that both groups of Danshen compounds may have anticancer effects (Tables 1 and 2). This systematic review provides an appraisal of the roles of reactive oxygen species (ROS) in cancer biology and anticancer therapy with Danshen. Based on the evidence demonstrating anticancer properties of Danshen and the roles of ROS in cancer biology, this review summarizes current data regarding the ROS-related anticancer effects of Danshen components and brings new insights for further anticancer research or clinical trials with this traditional Chinese herb.

## 2. Methods

Keyword searches were done using the combined terms “reactive oxygen species and cancer and Danshen” or “reactive oxygen species and cancer and* Salvia miltiorrhiza*”. These searches were done using the Medicine, PubMed, EMBASE, Cochrane library, CINAHL, and Scopus databases. The contents of the identified articles were summarized and the current review focused on the ROS-related anticancer effects of Danshen. After removing duplicate publications and excluding information that was unrelated to ROS, we collected 39 articles about the ROS-related anticancer effects of Danshen. These publications included a consideration of 34 lipophilic and 5 hydrophilic compounds isolated from Danshen.

## 3. Role of ROS in Cancer

Carcinogenesis is a progressive process from normal to cancerous cells. Reactive oxygen species (ROS) are closely related to carcinogenesis and play an important role in cancer. Previous studies have shown that ROS may be involved in multistep tumorigenesis including tumor initiation and transformation, tumor progression, tumor promotion, tumor angiogenesis, and tumor metastasis [56–58]. ROS are generated by both mitochondria and NADPH oxidases. Oxidative stress results from the generation of free radicals such as the superoxide anion, perhydroxyl radical, hydroxyl radical, and nitric oxide, as well as other nonradical but reactive species such as hydrogen peroxide, singlet oxygen, hypochlorous acid, and peroxynitrite [56, 57].

Mitochondria in malignant cells are characterized by the overproduction of ROS and differ structurally and functionally from those in normal cells [59]. A major source of ROS is oxidative metabolism in the mitochondria of eukaryotic cells. In normal cells, low-level concentrations of ROS, related to mitochondrial electron transport activity, are required for many cellular processes and signal transduction. Cancer cells generate more ROS as compared to normal cells. The increased generation of ROS in cancer cells may alter mitochondrial metabolism [59, 60] and disturb cellular signaling pathways [61, 62] that are mediated through the transcription factors NF-*κ*B and STAT3, hypoxia-inducible factor-1*α*, kinases, growth factors, cytokines, and other enzymes [63].

ROS can induce cellular DNA damage and DNA methylation [64] resulting in mutations, which causes healthy cells to transform into malignant cells. Some cancer cells overexpress the ROS-producing NADPH oxidases and ROS-removing antioxidant enzymes. Conversely, there is also evidence showing that excess ROS can result in cancer cell death through autophagy [65, 66] and/or apoptosis [62, 67]. Cancer cells may be more sensitive than normal cells to the overproduction of ROS. Thus, increasing oxidative stress by generating ROS exogenously may be selective for cancer cells without affecting normal cells [68, 69].

## 4. Lipophilic Components of Danshen

Tanshinone I, tanshinone IIA, acetyltanshinone IIA, cryptotanshinone, isocryptotanshinone, dihydrotanshinone, and miltirone are the main lipid-soluble potential anticancer constituents of Danshen. These compounds have shown anticancer activity (Table 1) with remarkable dose- and time-dependent inhibitory effects on the viability on prostate, lung, breast, leukemia, gastric, oral, colon, cervical, hepatoma, renal, melanoma, rhabdomyosarcoma, and neuroblastoma cancer cells. These effects, in terms of ROS, are described in more detail for each cell type in the following sections.

### 4.1. ROS-Related Anticancer Effects of Tanshinones on Prostate Cancer Cells

Tanshinone I enhanced tumor necrosis factor- (TNF-) related apoptosis inducing ligand (TRAIL) via increasing cleaved poly-ADP ribose polymerase (PARP), arresting cells in the subG1 phase, activating caspase-8 and caspase-9, and upregulating miR-135a-mediated death receptor 5 [17]. The induction of apoptosis and autophagy by tanshinone IIA was dependent on intracellular ROS production [20]. Cryptotanshinone suppressed androgen receptor-mediated cell growth [43] and induced ROS thereby phosphorylating (i.e., activating) P38/JNK and inhibiting Erk1/2, resulting in caspase-independent death in DU145 prostate cancer cells [40].

### 4.2. ROS-Related Anticancer Effects of Tanshinones on Lung Cancer Cells

Tanshinones inhibited the proliferation of 95D lung cancer cells by increasing caspase-3 activity and inducing apoptosis and prosurvival autophagy [16], through the increased generation of intracellular ROS. Tanshinone IIA decreased vascular endothelial growth factor/vascular endothelial growth factor receptor 2 (VEGF/VEGFR2) expression and induced apoptosis with cell cycle arrest at the S phase in human non-small cell lung cancer A549 cells [22]. These researchers noted that tanshinone IIA activated a ROS-induced, P53-independent [27], and caspase-dependent mitochondrial apoptotic cell death pathway that was characterized by an increased ratio of Bax to Bclx1 or Bax to Bcl-2, decreased mitochondrial membrane potential [30], caspase activation, PARP-1 cleavage, and cytochrome c release in A549 cells and small cell lung cancer H146 cells [31]. Cryptotanshinone also induced ROS-mediated prodeath autophagy through JNK signaling [37].

### 4.3. ROS-Related Anticancer Effects of Tanshinones on Breast Cancer Cells

Tanshinone I downregulated the PI3K/Akt/mTOR signaling pathway, induced cell cycle arrest, and inhibited the proliferation of breast cancer MCF-7 and MDA-MB-453 cells [18]. Acetyltanshinone IIA induced G1/S phase arrest and apoptosis with downregulation of receptor tyrosine kinases such as epidermal growth factor receptor (EGFR)/HER2 and activated AMP-activated protein kinase (AMPK) [33]. Acetyltanshinone IIA also induced ROS generation and Bax translocation to mitochondria resulting in mitochondrial damage, cytochrome c release, caspase-3 activation, and apoptotic cell death in HER2 positive breast cancer cells [34]. Cryptotanshinone suppressed estrogen receptor signaling and induced endoplasmic reticulum (ER) stress-mediated apoptosis [42] and ROS generation, activating P38/JNK and inhibiting Erk1/2. This led to caspase-independent cell death in MCF-7 breast cancer cells [40]. Isocryptotanshinone induced apoptosis and activated the mitogen-activated protein kinase (MAPK) signaling pathway in MCF-7 breast cancer cells [45].

### 4.4. ROS-Related Anticancer Effects of Tanshinones on Leukemia Cells

Some researchers found that tanshinone I activated caspase-3 and decreased human telomerase reverse transcriptase (hTERT) mRNA expression and telomerase activity, as well as downregulating survivin expression, in monocytic leukemia U937 THP-1 and SHI 1 cells [19]. Another study reported that tanshinone IIA induced apoptosis through the activation of PXR, which suppressed NF-*κ*B activity in leukemia U937 cells [29].

Cryptotanshinone inhibited cellular movement and induced G2/M phase arrest in acute lymphoblastic leukemia cells [36]. Another study revealed that cryptotanshinone enhanced TNF-*α*-induced apoptosis through the ROS-dependent activation of caspase-8 and p38 in chronic myeloid leukemia KBM-5 cells [44]. 15,16-Dihydrotanshinone I induced apoptosis through activation of the JNK and FasL signaling pathways in human HL-60 leukemia cells [48]. Miltirone induced G2/M cell cycle arrest and apoptosis in acute lymphoblastic leukemia cells [50].

### 4.5. ROS-Related Anticancer Effects of Tanshinones on Oral, Gastric, and Colon Cancer Cells

One previous study reported that tanshinone IIA induced apoptosis through the mitochondria-dependent pathway with the loss of mitochondrial membrane potential and the activation of caspase-9 and caspase-3 in human oral cancer KB cells [23]. Another study that examined the effects of tanshinone IIA reported that it suppressed cell growth by blocking glucose metabolism in gastric cancer cells [21]. Another article revealed that UDP- glucuronosyltransferase 1A compromised the apoptotic effects of tanshinone IIA by reducing its intracellular exposure and switching the NAD(P)H: quinine oxidoreductase 1-triggered redox cycle to metabolic elimination [24]. Other research noted that dihydrotanshinone I induced caspase and ROS-dependent apoptosis and autophagy in colon cancer cells [47].

### 4.6. ROS-Related Anticancer Effects of Tanshinones on Cervical Cancer Cells

Our previous studies showed that tanshinone IIA had anticancer effects on typical cervical HeLa and advanced cervical CaSki cancer cells. Tanshinone IIA induced apoptosis by interfering with the microtubule assembly process, leading to G2/M phase arrest and subsequent apoptosis in HeLa cells [32]. It also appeared to inhibit cell growth through activating the ER stress pathway and promoting caspase cascades with concomitant upregulation of the phosphorylation of the p38 and JNK-Bax-caspase-3/9 signaling pathways (Figure 3) in CaSki cells [25].

### 4.7. ROS-Related Anticancer Effects of Tanshinones on Hepatoma Cells

Tanshinone IIA increased Bax and caspase-3 levels and decreased CD31 expression in human hepatoma J5 cells [26]. Cryptotanshinone induced ER stress-mediated apoptosis [42] and induced G1 cell cycle arrest and autophagic cell death by activating the AMPK signaling pathway [38]. Dihydrotanshinone activated ROS-mediated phosphorylation of p38 MAPK in HepG2 cells [46]. Miltirone activated the caspase-dependent apoptotic pathway and triggered the ROS-mediated MAPK signaling pathway in human hepatoma HepG2 cells [49].

### 4.8. ROS-Related Anticancer Effects of Tanshinones on Renal Carcinoma Cells, Melanoma, Neuroblastoma, and Rhabdomyosarcoma Cells

Previous research noted that tanshinone IIA induced apoptosis in renal carcinoma cells by activating p53 expression and subsequently inducing the upregulation of p21 and Bax [28].

The other cryptotanshinone would restore the sensitivity of A375 melanoma cells that were resistant to TRAIL by upregulating the expression of death receptor 5 (DR5) [39]. It also could inhibit sodium nitroprusside-induced apoptosis by an antioxidant effect and by regulating NF-*κ*B and the MAPK pathway in Neuro-2a cells [41]. Cryptotanshinone was reported to induce ROS, then activate P38/JNK, and inhibit Erk1/2 in rhabdomyosarcoma cells. These effects then led to caspase-independent cell death in these cells [40].

## 5. ROS-Related Anticancer Effects of Hydrophilic Components Found in Danshen

Polyphenols, as dietary antioxidants, are most abundant in fruits, vegetables, and cereals [70, 71]. Numerous clinical studies, as well as* in vitro* and* in vivo* experiments, have strongly supported the ability of polyphenols to reduce the risk of many cancers. Some antioxidant polyphenols can downregulate TNF and might be useful as mitochondrially targeted anticancer drugs [72–74].

Salvianolic acids A and B are the main water-soluble polyphenolic derivatives found in Danshen. Similar to other natural polyphenols, they have potential anticancer effects (Table 2). Salvianolic acid A elevated ROS levels, downregulated P-glycoprotein, and triggered apoptosis by increasing caspase-3 activity and upregulating Bax expression, while downregulating Bcl-2 expression and disrupting the mitochondrial membrane potential in multidrug resistance MCF-7 human breast cancer cells [51]. Other research showed that salvianolic acids A and B had antioxidant and antiapoptotic properties that were involved in protecting SH-SY5Y human neuroblastoma cells against 1-methyl-4-phenylpyridinium ion-induced mitochondrial dysfunction. This dysfunction was characterized by loss of the mitochondrial membrane potential, condensation of nuclei, cytochrome c release, and increases in the Bax/Bcl-2 ratio [52, 54]. Salvianolic acid B prevented 6-hydroxydopamine-induced apoptosis in SH-SY5Y cells by reducing the increase of caspase-3 activity and the translocation of cytochrome c into the cytosol from mitochondria [55]. Another study revealed that salvianolic acid B induced apoptotic cell death in human glioma U87 cells through p53 and the phosphorylation and activation of p38 MAPK to increase ROS generation [53].

## 6. Conclusion

**Figure 2:**
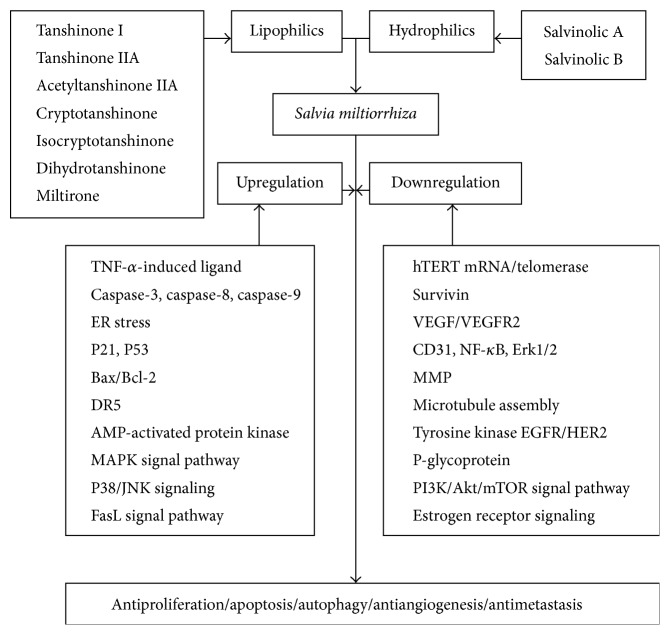
Schematic diagram of ROS-related anticancer effects mediated by Danshen. Upregulation: TNF-*α*, caspase-3, caspase-8, caspase-9, endoplasmic reticulum (ER) stress, P21, P53, Bax/Bcl-2, DR5, AMP-activated protein kinase, MAPK signaling pathways, the phosphorylation (activation) of p38/JNK signaling, and the FasL signaling pathway. Downregulation: hTERT mRNA, telomerase, survivin, VEGF/VEGFR2, CD31, NF-*κ*B, Erk1/2, MMP, microtubule assembly, tyrosine kinases such as EGFR/HER2, P-glycoprotein, and PI3K/Akt/mTOR, and estrogen receptor signaling.

**Figure 3:**
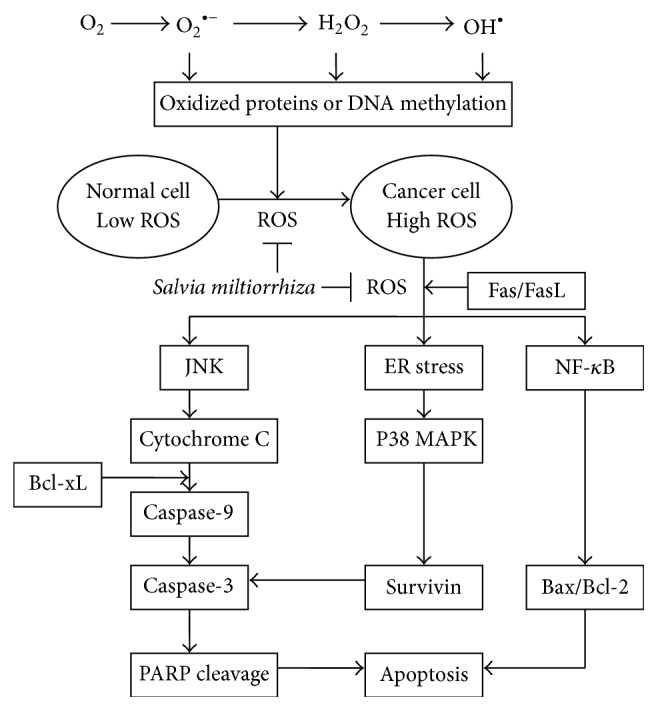
Schematic diagram of effects of* Salvia miltiorrhiza* on reactive oxygen species-related apoptosis of cancer cells.

Danshen may be a potential complementary or alternative therapy for various cancer patients. We found the potential utility of this natural product, or its active constituents including lipophilic compounds such as tanshinone I, tanshinone IIA, acetyltanshinone IIA, cryptotanshinone, isocryptotanshinone, dihydrotanshinone, 15,16-dihydrotanshinone I, miltirone, and hydrophilic phenolic acids such as salvianolic acids A and B (Figure 1). The ROS-related anticancer effects of the lipophilic and hydrophilic constituents isolated from Danshen vary, depending on the specific type of cancer cells (Tables 1 and 2). Overall, Danshen can suppress cell proliferation through antioxidation on tumor initiation and induce apoptosis (Figure 3) or autophagy through ROS generation on tumor progression, tumor promotion, and tumor metastasis. Some components of Danshen may enhance TNF-*α*-induced apoptosis and upregulate caspase-3, caspase-8, caspase-9, ER stress, P21, P53, Bax/Bcl-2, DR5, and AMPK and activate the p38/JNK, MAPK, or FasL signaling pathways. Conversely, these compounds can downregulate hTERT mRNA, telomerase, survivin, VEGF/VEGFR2, CD31, NF-*κ*B, Erk1/2, MMPs, microtubule assembly, tyrosine kinases such as EGFR/HER2 and P-glycoprotein and inhibit the PI3K/Akt/mTOR or estrogen receptor signaling pathways (Figure 2). Combined, these effects inhibit cancer cell proliferation by arresting cell cycle progression, inducing cancer cell apoptosis and/or autophagy, and exerting antiangiogenic and antimetastatic effects. However, in accordance with laboratory evidences obtained* in vitro* and* in vivo*, rigorous human studies are needed to demonstrate the anticancer effects of Danshen. Future well-designed clinical studies, such as randomized controlled clinical trials, will be necessary to confirm the efficacy of Danshen as an anticancer agent in human patients.

## Figures and Tables

**Table 1:** Lipophilic components from *Salvia miltiorrhiza* that modify ROS-related effects on cancer cells.

Components [reference]	Cancer cells	Effects
Tanshinones [16]	Lung cancer 95D cells	Induces apoptosis and prosurvival autophagy mediated by increasing the formation of intracellular ROS

Tanshinone I [17]	Prostate cancer cells	Enhances TRAIL via upregulation of miR-135a-3p-mediated death receptor 5

Tanshinone I [18]	Human breast cancer MDA-MB-453 cells	Induces antiproliferative activity and cell cycle arrest by inhibiting the PI3K/Akt/mTOR signaling pathways

Tanshinone I [19]	Leukemia U937 THP-1 and SHI 1 cells	Induces apoptosis by activating caspase-3 and decreasing hTERT mRNA expression and telomerase activity, as well as downregulating survivin expression

Tanshinone IIA [20]	Prostate cancer cells	Induces apoptosis and autophagy that depends on intracellular ROS production

Tanshinone IIA [21]	Gastric cancer cells	Suppresses cell growth by blocking glucose metabolism

Tanshinone IIA [22]	Human non-small cell lung cancer A549 cells	Decreases VEGF/VEGFR2 expression and induces apoptosis and cell cycle arrest at the S phase

Tanshinone IIA [23]	Human oral cancer KB cells	Induces apoptosis through the mitochondria-dependent pathway in which there is a loss of the mitochondrial membrane potential and activation of caspase-3 and caspase-9

Tanshinone IIA [24]	Human colon cancer cells	UDP-glucuronosyltransferase 1A compromises the intracellular accumulation and resultant apoptotic effect of tanshinone IIA

Tanshinone IIA [25]	Cervical cancer CaSki cells	Inhibits cell growth by activating ER stress pathways and promoting caspase cascades with concomitant upregulation of p38 and JNK phosphorylation and signaling

Tanshinone IIA [26]	Human hepatoma J5 cells	Increases Bax and caspase-3 and decreases CD31 expression

Tanshinone IIA [27]	Non-small cell lung cancer H596 cells	Activates ROS-triggered, p53-independent, and caspase-dependent mitochondrial apoptotic cell death pathway

Tanshinone IIA [28]	786-O human renal cell carcinoma cells	Induces apoptosis by activating p53 expression and subsequently upregulating p21 and Bax

Tanshinone IIA [29]	Leukemia U937 cells	Induces apoptosis by activating PXR, which suppresses the activity of NF-*κ*B

Tanshinone IIA [30]	human non-small lung cancer A549 cells	Induces apoptosis by increasing ROS and the ratio of Bax/Bcl-2 and then decreasing the mitochondrial membrane potential, which leads to cytochrome c release

Tanshinone IIA [31]	Small cell lung cancer H146 cells	Inhibits cell growth by upregulating the Bax/Bcl-2 ratio and decreasing the mitochondrial membrane potential

Tanshinone IIA [32]	Cervical cancer HeLa cells	Inhibits cell growth by interfering with the process of microtubule assembly, leading to G2/M phase arrest and subsequent apoptosis

Acetyltanshinone IIA [33]	Breast cancer	Induces G1/S phase arrest and apoptosis by downregulating the receptor tyrosine kinases EGFR/HER2 and activating AMP-activated protein kinase

Acetyltanshinone IIA [34]	Breast cancer cells	Induces ROS generation and Bax translocation to mitochondria, resulting in mitochondrial damage, cytochrome c release, caspase-3 activation, and apoptotic cell death

Cryptotanshinone [35]	Breast cancer cells	Suppresses estrogen receptor signaling

Cryptotanshinone [36]	Acute lymphoblastic leukemia cells	Inhibits cellular movement and induces G2/M cell cycle arrest and apoptosis

Cryptotanshinone [37]	Lung cancer cells	Induces prodeath autophagy through JNK signaling that is mediated by ROS generation

Cryptotanshinone [38]	HepG2 hepatoma	Induces G1 cell cycle arrest and autophagic cell death by activating the AMP-activated protein kinase signaling pathway

Cryptotanshinone [39]	A375 melanoma cells	Restores sensitivity in cancer cells that are resistant to TRAIL by upregulating DR5 expression

Cryptotanshinone [40]	Rh30 human rhabdomyosarcoma; DU145 prostate carcinoma; and human MCF-7 breast cancer cells	Induces ROS, thereby activating p38/JNK and inhibiting Erk1/2 leading to caspase-independent cell death

Cryptotanshinone [41]	Neuro-2a cells	Inhibits sodium nitroprusside-induced apoptosis by antioxidant effects and regulating the NF-*κ*B and MAPK pathways

Cryptotanshinone [42]	HepG2 hepatoma and MCF-7 breast cancer cells	Induces ER stress-mediated apoptosis

Cryptotanshinone [43]	Prostate cancer cells	Suppresses androgen receptor- (AR-) mediated growth by blocking AR dimerization and formation of the AR-coregulator complex

Cryptotanshinone [44]	Chronic myeloid leukemia KBM-5 cells	Enhances TNF-*α*-induced apoptosis through ROS-dependent activation of caspase-8 and p38

Isocryptotanshinone [45]	Human breast cancer MCF-7 cells	Induces apoptosis and activates MAPK signaling pathways

Dihydrotanshinone [46]	HepG2 cells	Activates ROS-mediated phosphorylation of p38 MAPK

Dihydrotanshinone I [47]	Colon cancer	Induces caspase- and ROS-dependent apoptosis and autophagy

15,16-Dihydrotanshinone I [48]	Human HL-60 Leukemia Cells	Induces apoptosis through activation of the JNK and FasL signaling pathways

Miltirone [49]	Human hepatoma HepG2 cells	Activates caspase-dependent apoptotic pathways and triggers ROS-mediated MAPK signaling pathways

Miltirone [50]	Acute lymphoblastic leukemia cells	Induces G2/M cell cycle arrest and apoptosis

**Table 2:** Hydrophilic components from *Salvia miltiorrhiza* that modify ROS-related effects on cancer cells.

Components [reference]	Cancer cells	Effects
Salvianolic acid A [51]	MCF-7 breast cancer cells	Downregulates the level of P-glycoprotein and triggers apoptosis, which is associated with increased caspase-3 activity, disrupted mitochondrial membrane potential, downregulated Bcl-2 expression, and upregulated Bax expression in resistant cells

Salvianolic acid A [52]	Human neuroblastoma SH-SY5Y cells	Prevents 1-methyl-4-phenylpyridinium ion-induced cytotoxicity, which may be ascribed to its antioxidant properties and antiapoptotic activity via regulating the expression of Bcl-2 and Bax

Salvianolic acid B [53]	Human glioma U87 cells	Induces apoptosis through p38-mediated ROS generation

Salvianolic acid B [54]	Human neuroblastoma SH-SY5Y cells	Prevents 1-methyl-4-phenylpyridinium-induced apoptosis by relieving oxidative stress and modulating the apoptotic process

Salvianolic acid B [55]	Human neuroblastoma SH-SY5Y cells	Prevents dopamine-induced apoptosis that may be mediated by the ROS and the Erk and Bcl-2 pathways

## References

[1] Parekh H. S., Liu G., Wei M. Q. (2009). A new dawn for the use of traditional Chinese medicine in cancer therapy. *Molecular Cancer*.

[2] Wu T., Ni J., Wu J. (2008). Danshen (Chinese medicinal herb) preparations for acute myocardial infarction. *Cochrane Database of Systematic Reviews*.

[3] Wu B., Liu M., Zhang S. (2007). Dan Shen agents for acute ischaemic stroke. *Cochrane Database of Systematic Reviews*.

[4] Luo J., Song W., Yang G., Xu H., Chen K. (2015). Compound danshen (*Salvia miltiorrhiza*) dripping pill for coronary heart disease: an overview of systematic reviews. *The American Journal of Chinese Medicine*.

[5] Liu Y., Huang Y., Zhao C. (2014). *Salvia miltiorrhiza* injection on pulmonary heart disease: a systematic review and meta-analysis. *The American Journal of Chinese Medicine*.

[6] Guo Y., Li Y., Xue L. (2014). Salvia miltiorrhiza: an ancient Chinese herbal medicine as a source for anti-osteoporotic drugs. *Journal of Ethnopharmacology*.

[7] Hügel H. M., Jackson N. (2014). Danshen diversity defeating dementia. *Bioorganic & Medicinal Chemistry Letters*.

[8] Chen J., Lv Q., Yu M., Zhang X., Gou J. (2010). Randomized clinical trial of Chinese herbal medications to reduce wound complications after mastectomy for breast carcinoma. *British Journal of Surgery*.

[9] Cho J. H., Cho C. K., Shin J. W., Son J. Y., Kang W., Son C. G. (2009). Myelophil, an extract mix of Astragali Radix and Salviae Radix, ameliorates chronic fatigue: a randomised, double-blind, controlled pilot study. *Complementary Therapies in Medicine*.

[10] Bao Y. X., Wong C. K., Leung S. F. (2006). Clinical studies of immunomodulatory activities of Yunzhi-Danshen in patients with nasopharyngeal carcinoma. *Journal of Alternative and Complementary Medicine*.

[11] Wong C. K., Bao Y. X., Wong E. L., Leung P. C., Fung K. P., Lam C. W. (2005). Immunomodulatory activities of Yunzhi and Danshen in post-treatment breastcancer patients. *The American Journal of Chinese Medicine*.

[12] Chen X., Guo J., Bao J., Lu J., Wang Y. (2014). The anticancer properties of *Salvia miltiorrhiza* Bunge (Danshen): a systematic review. *Medicinal Research Reviews*.

[13] Cai Y., Zhang W., Chen Z., Shi Z., He C., Chen M. (2016). Recent insights into the biological activities and drug delivery systems of tanshinones. *International Journal of Nanomedicine*.

[14] Zhang Y., Jiang P., Ye M., Kim S.-H., Jiang C., Lü J. (2012). Tanshinones: sources, pharmacokinetics and anti-cancer activities. *International Journal of Molecular Sciences*.

[15] Wang X., Morris-Natschke S. L., Lee K.-H. (2007). New developments in the chemistry and biology of the bioactive constituents of Tanshen. *Medicinal Research Reviews*.

[16] Gao H., Sun W., Zhao W. (2015). Total tanshinones-induced apoptosis and autophagy via reactive oxygen species in lung cancer 95D Cells. *American Journal of Chinese Medicine*.

[17] Shin E. A., Sohn E. J., Won G. (2014). Upregulation of microRNA135a-3p and death receptor 5 plays a critical role in Tanshinone I sensitized prostate cancer cells to TRAIL induced apoptosis. *Oncotarget*.

[18] Wang L., Wu J., Lu J., Ma R., Sun D., Tang J. (2015). Regulation of the cell cycle and PI3K/Akt/mTOR signaling pathway by tanshinone I in human breast cancer cell lines. *Molecular Medicine Reports*.

[19] Liu X. D., Fan R. F., Zhang Y. (2010). Down-regulation of telomerase activity and activation of caspase-3 are responsible for tanshinone i-induced apoptosis in monocyte leukemia cells *in Vitro*. *International Journal of Molecular Sciences*.

[20] Li C., Han X., Zhang H., Wu J., Li B. (2016). The interplay between autophagy and apoptosis induced by tanshinone IIA in prostate cancer cells. *Tumor Biology*.

[21] Lin L., Hsia C., Hsu C., Huang H., Juan H. (2015). Integrating transcriptomics and proteomics to show that tanshinone IIA suppresses cell growth by blocking glucose metabolism in gastric cancer cells. *BMC Genomics*.

[22] Xie J., Liu J., Liu H. (2015). The antitumor effect of tanshinone IIA on anti-proliferation and decreasing VEGF/VEGFR2 expression on the human non-small cell lung cancer A549 cell line. *Acta pharmaceutica Sinica B*.

[23] Tseng P.-Y., Lu W.-C., Hsieh M.-J., Chien S.-Y., Chen M.-K. (2014). Tanshinone IIA induces apoptosis in human oral cancer KB cells through a mitochondria-dependent pathway. *BioMed Research International*.

[24] Liu M., Wang Q., Liu F. (2013). UDP-glucuronosyltransferase 1A compromises intracellular accumulation and anti-cancer effect of tanshinone IIA in human colon cancer cells. *PLoS ONE*.

[25] Pan T.-L., Wang P.-W., Hung Y.-C., Huang C.-H., Rau K.-M. (2013). Proteomic analysis reveals tanshinone IIA enhances apoptosis of advanced cervix carcinoma CaSki cells through mitochondria intrinsic and endoplasmic reticulum stress pathways. *Proteomics*.

[26] Chien S.-Y., Kuo S.-J., Chen Y.-L., Chen D.-R., Cheng C.-Y., Su C.-C. (2012). Tanshinone IIA inhibits human hepatocellular carcinoma J5 cell growth by increasing Bax and caspase 3 and decreasing CD31 expression in vivo. *Molecular Medicine Reports*.

[27] Liu F., Yu G., Wang G. (2012). An NQO1-initiated and p53-independent apoptotic pathway determines the anti-tumor effect of tanshinone IIA against non-small cell lung cancer. *PLoS ONE*.

[28] Wei X., Zhou L., Hu L., Huang Y. (2012). Tanshinone IIA arrests cell cycle and induces apoptosis in 786-O human renal cell carcinoma cells. *Oncology Letters*.

[29] Liu C., Li J., Wang L. (2012). Analysis of tanshinone IIA induced cellular apoptosis in leukemia cells by genome -wide expression profiling. *BMC Complementary and Alternative Medicine*.

[30] Chiu T.-L., Su C.-C. (2010). Tanshinone IIA induces apoptosis in human lung cancer A549 cells through the induction of reactive oxygen species and decreasing the mitochondrial membrane potential. *International Journal of Molecular Medicine*.

[31] Cheng C.-Y., Su C.-C. (2010). Tanshinone IIA may inhibit the growth of small cell lung cancer H146 cells by up-regulating the Bax/Bcl-2 ratio and decreasing mitochondrial membrane potential. *Molecular Medicine Reports*.

[32] Pan T.-L., Hung Y.-C., Wang P.-W. (2010). Functional proteomic and structural insights into molecular targets related to the growth inhibitory effect of tanshinone IIA on HeLa cells. *Proteomics*.

[33] Guerram M., Jiang Z.-Z., Yousef B. A. (2015). The potential utility of acetyltanshinone IIA in the treatment of HER2-overexpressed breast cancer: induction of cancer cell death by targeting apoptotic and metabolic signaling pathways. *Oncotarget*.

[34] Tian H.-L., Yu T., Xu N.-N. (2010). A novel compound modified from tanshinone inhibits tumor growth in vivo via activation of the intrinsic apoptotic pathway. *Cancer Letters*.

[35] Li S., Wang H., Hong L. (2015). Cryptotanshinone inhibits breast cancer cell growth by suppressing estrogen receptor signaling. *Cancer Biology & Therapy*.

[36] Wu C.-F., Klauck S. M., Efferth T. (2015). Anticancer activity of cryptotanshinone on acute lymphoblastic leukemia cells. *Archives of Toxicology*.

[37] Hao W., Zhang X., Zhao W. (2015). Cryptotanshinone induces pro-death autophagy through JNK signaling mediated by reactive oxygen species generation in lung cancer cells. *Anti-Cancer Agents in Medicinal Chemistry*.

[38] Park I.-J., Yang W. K., Nam S.-H. (2014). Cryptotanshinone induces G1 cell cycle arrest and autophagic cell death by activating the AMP-activated protein kinase signal pathway in HepG2 hepatoma. *Apoptosis*.

[39] Tse A. K.-W., Chow K.-Y., Cao H.-H. (2013). The herbal compound cryptotanshinone restores sensitivity in cancer cells that are resistant to the tumor necrosis factor-related apoptosis-inducing ligand. *Journal of Biological Chemistry*.

[40] Chen W., Liu L., Luo Y. (2012). Cryptotanshinone activates p38/JNK and inhibits Erk1/2 leading to caspase-independent cell death in tumor cells. *Cancer Prevention Research*.

[41] Mahesh R., Jung H. W., Kim G. W., Kim Y. S., Park Y.-K. (2012). Cryptotanshinone from *Salviae miltiorrhizae* radix inhibits sodium-nitroprusside-induced apoptosis in neuro-2a cells. *Phytotherapy Research*.

[42] Park I.-J., Kim M.-J., Park O. J. (2012). Cryptotanshinone induces ER stress-mediated apoptosis in HepG2 and MCF7 cells. *Apoptosis*.

[43] Xu D., Lin T.-H., Li S. (2012). Cryptotanshinone suppresses androgen receptor-mediated growth in androgen dependent and castration resistant prostate cancer cells. *Cancer Letters*.

[44] Kim J.-H., Jeong S.-J., Kwon T.-R. (2011). Cryptotanshinone enhances TNF-*α*-induced apoptosis in chronic myeloid leukemia KBM-5 cells. *Apoptosis*.

[45] Zhang X., Luo W., Zhao W., Lu J., Chen X. (2015). Isocryptotanshinone induced apoptosis and activated MAPK signaling in human breast cancer MCF-7 cells. *Journal of Breast Cancer*.

[46] Lee W. Y., Liu K. W., Yeung J. H. (2009). Reactive oxygen species-mediated kinase activation by dihydrotanshinone in tanshinones-induced apoptosis in HepG2 cells. *Cancer Letters*.

[47] Wang L., Hu T., Shen J. (2015). Dihydrotanshinone I induced apoptosis and autophagy through caspase dependent pathway in colon cancer. *Phytomedicine*.

[48] Liu J.-J., Wu H.-H., Chen T.-H., Leung W., Liang Y.-C. (2015). 15,16-Dihydrotanshinone I from the functional food *Salvia miltiorrhiza* exhibits anticancer activity in human HL-60 leukemia cells: in vitro and in vivo studies. *International Journal of Molecular Sciences*.

[49] Zhou X., Wang Y., Lee W. Y. (2015). Miltirone is a dual inhibitor of p-glycoprotein and cell growth in doxorubicin-resistant HepG2 cells. *Journal of Natural Products*.

[50] Wu C.-F., Efferth T. (2015). Miltirone induces G2/M cell cycle arrest and apoptosis in CCRF-CEM acute lymphoblastic leukemia cells. *Journal of Natural Products*.

[51] Wang X., Wang C., Zhang L. (2015). Salvianolic acid A shows selective cytotoxicity against multidrug-resistant MCF-7 breast cancer cells. *Anti-Cancer drugs*.

[52] Wang X.-J., Xu J.-X. (2005). Salvianic acid A protects human neuroblastoma SH-SY5Y cells against MPP+-induced cytotoxicity. *Neuroscience Research*.

[53] Wang Z.-S., Luo P., Dai S.-H., Liu Z.-B., Zheng X.-R., Chen T. (2013). Salvianolic acid b induces apoptosis in human glioma U87 cells through p38-mediated ROS generation. *Cellular and Molecular Neurobiology*.

[54] Zeng G., Tang T., Wu H.-J. (2010). Salvianolic acid B protects SH-SY5Y neuroblastoma cells from 1-methyl-4-phenylpyridinium-induced apoptosis. *Biological and Pharmaceutical Bulletin*.

[55] Tian L.-L., Wang X.-J., Sun Y.-N. (2008). Salvianolic acid B, an antioxidant from Salvia miltiorrhiza, prevents 6-hydroxydopamine induced apoptosis in SH-SY5Y cells. *International Journal of Biochemistry and Cell Biology*.

[56] Sosa V., Moliné T., Somoza R., Paciucci R., Kondoh H., LLeonart M. E. (2013). Oxidative stress and cancer: an overview. *Ageing Research Reviews*.

[57] Kryston T. B., Georgiev A. B., Pissis P., Georgakilas A. G. (2011). Role of oxidative stress and DNA damage in human carcinogenesis. *Mutation Research*.

[58] Wu W. S. (2010). The signaling mechanism of ROS in tumor progression. *Autophagy*.

[59] Yang Y., Karakhanova S., Hartwig W. (2016). Mitochondria and mitochondrial ROS in cancer: novel targets for anticancer therapy. *Journal of Cellular Physiology*.

[60] Sabharwal S. S., Schumacker P. T. (2014). Mitochondrial ROS in cancer: initiators, amplifiers or an Achilles' heel?. *Nature Reviews Cancer*.

[61] Bauer G. (2014). Targeting extracellular ROS signaling of tumor cells. *Anticancer Research*.

[62] Wu C. C., Bratton S. B. (2013). Regulation of the intrinsic apoptosis pathway by reactive oxygen species. *Antioxidants & Redox Signaling*.

[63] Prasad S., Gupta S. C., Tyagi A. K. (2016). Reactive oxygen species (ROS) and cancer: role of antioxidative nutraceuticals. *Cancer Letters*.

[64] Wu Q., Ni X. (2015). ROS-mediated DNA methylation pattern alterations in carcinogenesis. *Current Drug Targets*.

[65] Li L., Ishdorj G., Gibson S. B. (2012). Reactive oxygen species regulation of autophagy in cancer: implications for cancer treatment. *Free Radical Biology and Medicine*.

[66] Dewaele M., Maes H., Agostinis P. (2006). ROS-mediated mechanisms of autophagy stimulation and their relevance in cancer therapy. *Cancer and Metastasis Reviews*.

[67] Yang Y., Karakhanova S., Werner J., Bazhin A. V. (2013). Reactive oxygen species in cancer biology and anticancer therapy. *Current Medicinal Chemistry*.

[68] Gupta R. K., Patel A. K., Shah N. (2014). Oxidative stress and antioxidants in disease and cancer: a review. *Asian Pacific Journal of Cancer Prevention*.

[69] Liu J., Wang Z. (2015). Increased oxidative stress as a selective anticancer therapy. *Oxidative Medicine and Cellular Longevity*.

[70] Pandey K. B., Rizvi S. I. (2009). Plant polyphenols as dietary antioxidants in human health and disease. *Oxidative Medicine and Cellular Longevity*.

[71] Harasym J., Oledzki R. (2014). Effect of fruit and vegetable antioxidants on total antioxidant capacity of blood plasma. *Nutrition*.

[72] Gorlach S., Fichna J., Lewandowska U. (2015). Polyphenols as mitochondria-targeted anticancer drugs. *Cancer Letters*.

[73] Nichenametla S. N., Taruscio T. G., Barney D. L., Exon J. H. (2006). A review of the effects and mechanisms of polyphenolics in cancer. *Critical Reviews in Food Science and Nutrition*.

[74] Szliszka E., Krol W. (2011). The role of dietary polyphenols in tumor necrosis factor-related apoptosis inducing ligand (TRAIL)-induced apoptosis for cancer chemoprevention. *European Journal of Cancer Prevention*.

